# Contributions of Work-to-Family Enrichment to Parental Food Monitoring and Satisfaction with Food-Related Life during the COVID-19 Pandemic in Dual-Earner Parents and Their Adolescent Children

**DOI:** 10.3390/nu14194140

**Published:** 2022-10-05

**Authors:** Berta Schnettler, Ligia Orellana, Edgardo Miranda-Zapata, Mahia Saracostti, Héctor Poblete, Germán Lobos, Cristian Adasme-Berríos, María Lapo, Katherine Beroíza, Klaus G. Grunert

**Affiliations:** 1 Facultad de Ciencias Agropecuarias y Medioambiente, Universidad de La Frontera, Temuco 4780000, Chile; 2 Scientific and Technological Bioresource Nucleus (BIOREN-UFRO), Universidad de La Frontera, Temuco 4780000, Chile; 3 Centro de Excelencia en Psicología Económica y del Consumo, Núcleo de Ciencias Sociales, Universidad de La Frontera, Temuco 4780000, Chile; 4 Facultad de Especialidades Empresariales, Universidad Católica de Santiago de Guayaquil, Guayaquil 090150, Ecuador; 5 Centro de Investigación Escolar y Desarrollo (Cied-UCT), Facultad de Educación, Universidad Católica de Temuco, Temuco 4780000, Chile; 6 Department of Psychology, Universidad Autónoma de Chile, Temuco 4780000, Chile; 7 Escuela de Trabajo Social, Universidad de Valparaíso, Valparaíso 2340000, Chile; 8 Facultad de Economía y Negocios, Universidad de Talca, Talca 3460000, Chile; 9 Departamento de Economía y Administración, Universidad Católica del Maule, Talca 3460000, Chile; 10 MAPP Centre, Aarhus University, 8000 Aarhus, Denmark; 11 School of Marketing and Communication, University of Vaasa, 65100 Vaasa, Finland

**Keywords:** work resources, enrichment, food parenting practices, monitoring, satisfaction with food-related life, dual-earner couples, adolescents

## Abstract

Evidence shows that numerous family-related variables influence parents’ use of different food parenting practices (FPP), but less is known about the influence of parents’ work-related variables on their use of FPP, and their own and their children’s outcomes in the food domain. To fill this gap, the present study explored intra-individual and inter-individual effects between work-to-family enrichment (WtoFE), parents’ monitoring practices, the adolescent’s perception of their parents’ monitoring practices, and the three family members’ satisfaction with food-related life (SWFoL), in different-sex dual-earner parents with adolescent children. The mediating role of monitoring between WtoFE and SWFoL was also tested. A sample of 430 different-sex dual-earner parents and one of their adolescent children (average age 13.0 years, 53.7% female) were recruited in Rancagua, Chile, during March and June 2020. The three family members answered the monitoring dimension of the Compressive Feeding Practices Questionnaire and the Satisfaction with Food-Related Life Scale. Parents answered a measure of WtoFE based on the Work–Home Interaction Survey. Analyses were conducted using the Actor–Partner Interdependence Model and structural equation modelling. Results showed a positive association between WtoFE and SWFoL, directly (*p* < 0.001) and through monitoring in fathers (95% confidence interval [0.010, 0.097], actor effect). The father’s (*p* = 0.042) and mother’s (*p* = 0.006) WtoFE was positively associated with their adolescent’s SWFoL (partner effects). The father’s (*p* = 0.002) and mother’s (*p* = 0.036) WtoFE were positively associated with their own monitoring (actor effect), while only the father’s WtoFE (*p* = 0.014) was positively associated with the adolescent’s perception of their parents’ monitoring (partner effect). The father’s (*p* = 0.018) and mother’s (*p* = 0.003) monitoring, as well as the adolescents’ perception of their parents’ monitoring (*p* = 0.033), were positively associated with their own SWFoL (actor effects), while the mother’s monitoring (*p* = 0.043) was also associated with the father’s SWFoL (partner effects). Findings suggest that both parents’ WtoFE improved their monitoring practices, which, in turn, improved their own SWFoL and their adolescent child’s SWFoL. Policymakers and organizations must aim to promote the WtoFE of working parents.

## 1. Introduction

The COVID-19 pandemic, declared in March 2020, led to establishing individual and collective measures to contain the spread of the virus. Perhaps the most influential of these measures was lockdown mandates across the globe, which disrupted everyday activities in all life spheres [1]. Lockdown measures entailed that families stayed at home, which enforced changes in household functioning, including patterns related to eating habits [2,3,4]. Parents who kept their job during the pandemic faced challenges [5] regarding the balance between working from home, childcare and other household responsibilities [4,6]. These challenges have been related to higher stress in working parents, but they also could coexist with positive consequences of the pandemic, such as families could spend more time together and were more likely to have frequent family meals [3,4,7]. These positive spillovers from work life to family life [8,9] might influence the way in which parents try to encourage healthy eating in their children, by applying food parenting practices.

Food parenting practices (FPP) are behaviors or techniques that parents use to influence what, when, or how much a child eats, to promote healthy eating and prevent overweight and obesity [10]. FPP, however, have been related to both positive and negative outcomes regarding children’s diet quality, nutritional status and well-being [11,12,13]. According to Vaughn et al. [14], there are three higher-order FPP constructs: coercive control (e.g., restriction, pressure to eat), structure (e.g., rules and limits around food, monitoring, meal and snack routines), and autonomy, support or promotion (e.g., nutrition education, child involvement). Some studies have examined the use of FPP during the COVID-19 pandemic in samples of parents with young children [4,7]. However, research on the use of FPP on adolescents during the pandemic is still limited [4]. 

In the food domain, adolescents pose different challenges for parents than younger children. Parents typically remain responsible for feeding their adolescent children [15,16], but the latter are at a developmental stage that entails achieving autonomy [11,17], and this increased independence in decision-making includes their food and mealtimes [18]. As adolescents have a less developed understanding of the negative effects of unhealthy eating than their parents [19], they show a strong preference for tasty but unhealthy foods [20] and a limited concern about future health [18]. Moreover, dietary behaviors tend to worsen as children transition to adolescence, leading to increased risk for weight gain [11,21]. Therefore, this transitional period is critical for addressing eating behaviors [11], as food preferences and behaviors formed during adolescence tend to be maintained into adulthood [17]. Adolescents, thus, still need their parents to act as role models, guides and disciplinarians of dietary habits [15]. Among the FPP used by parents of adolescents, monitoring [11,19,22,23,24,25] is defined as the frequency with which parents keep track of their child’s intake of unhealthy foods [26].

Research on antecedents of FPP have focused on family-related variables, such as parents’ and children’s weight status and eating behaviors, age of children, family size, family income or socioeconomic level and family structure, e.g., [21,23,24,27,28,29]; less is known about work-related variables that may influence parents’ use of FPP and their effect on children’s outcomes. An effect of work-related variables can be expected, because workers’ high job demands have been associated with lower diet quality for themselves and their families [30,31]. This relationship occurs because personal resources such as time and energy that are invested in workplace responsibilities, are not available for food preparation and consumption, e.g., [30,31]. However, work–family dynamics entail not only job demands that may adversely affect family life, but possibly also the acquisition of resources that can be invested to benefit family life [32]. To explore these resource gains, Greenhaus and Powell [33] developed the concept of work-to-family enrichment (WtoFE) to refer to the contributions that work life can make to improve family life. 

This study focuses on WtoFE, that is, the transmission of resources (i.e., social, psychological, material, and emotional resources) from the workplace to the family/home domain [33]. This resource transmission can, in turn, lead to higher well-being in the family domain for both workers and their families [32,34,35], as resources gained in the work domain can also crossover from workers to their family members [36]. In addition, WtoFE is consistent with the work–home resource model (W-HR, [37]), which proposes that activities performed in the work domain can lead to the development of personal resources that can be invested in the home domain. In this sense, WtoFE can enhance satisfaction in other domains of life beyond family and work (e.g., [34]), such as the food domain [38], as work resources can facilitate a more efficient performance of food-related tasks [37]. Based on reports of a positive relationship between food and work domains in dual-earner couples [38,39,40], a resource exchange between these domains can be expected. The impact of this exchange might be observed in satisfaction with food-related life (SWFoL, [41]), which comprises a person’s overall cognitive assessment of their food and eating habits—including meal planning, shopping and meal preparation, consumption, and disposal. 

Studies conducted in the European Union provide evidence in favor of both an intra-individual (actor effects) and inter-individual (partner effects) transmission of work resources within family members [35,42,43]. In the food domain, evidence shows that resources provided by a high work–home balance positively influence the male’s SWFoL, but also their female partner’s SWFoL, and vice-versa in dual-earner couples [44]; and that mothers’ and fathers’ higher balance between their work and home lives has a positive influence on their adolescents’ SWFoL [45]. In addition, a recent study showed a positive association between WtoFE and SWFoL in mothers and fathers, while both parents’ WtoFE was indirectly associated with their adolescents’ SWFoL [38]. Furthermore, it has been reported that fathers’ perceived workplace support for families positively affected their own diet quality as well as the mothers’ and their adolescents’ diet quality [40]. As higher levels of SWFoL have been related to healthier diets in adult and adolescent samples [20,21,46], we argue that workers’ WtoFE may improve their performance in the food domain (i.e., preparing healthy meals), which, in turn, may lead to higher SWFoL in workers (actor effects), their working partners and adolescent children (crossover or partner effects). 

Researchers have reported that parents’ WtoFE is associated with more positive parent–child interactions and the parental role [43,47,48,49,50]. Although no previous studies have evaluated the relationship between a parent’s WtoFE and their FPP, some studies have shown that parents with higher balance between their work and home-related tasks tend to be more involved in their children’s food habits [51], and are more likely to have frequent family meals and serve healthier meals to their families [52,53]. In addition, a qualitative study showed that both mothers and fathers in dual-earner couples are concerned about having enough resources (e.g., money, time, skills) to provide healthy foods to their family members, including the spouse [20]. Therefore, we argue that both parents’ WtoFE may improve their performance in the food domain by exerting monitoring practices in their children, which may also cross over to the other parent and their adolescent child, as mirrored in their perception of their parents’ monitoring practices. 

Little is known about the outcomes of different food-related parenting practices on adolescents’ and their parents’ well-being, but studies indicate that the FPP exerted by parents may have a positive or a negative influence in this regard [11,16]. There is evidence of a positive relationship between parents’ monitoring practices and positive diet outcomes in adolescents. Studies in Norway [19], the United States [23,25], and Spain [22] report a positive relationship between parents’ monitoring and healthy eating in adolescents aged 10 to 17. In addition, healthier diets have been associated with greater levels of SWFoL in both adult and adolescent samples [20,21,46]. Therefore, we expect that both the mother’s and father’s monitoring, and the adolescent’s perception of their parents’ monitoring practices, are linked to higher levels of SWFoL in the three family members. 

At the same time, positive parental monitoring in food consumption supports an adolescent’s perception that parents want them to make healthy food choices to prevent health issues, such as those related to overweight and obesity [25]. While there is evidence that controlling parenting practices may be associated with higher levels of anxiety and depression in adolescents [54], other studies [16,21,29] have reported that maternal control of child snacking behavior is associated with both the mother’s and their adolescent child’s SWFoL and life satisfaction (i.e., the assessment one makes regarding one’s overall life or specific life domains [55]). Taken together, these results suggest that positive FPP have beneficial influences on both the parent and the child through crossover effects. These crossover effects may also be expected from one parent to the other. 

Studies linking WtoFE and satisfaction in parents and their children are scarce [56]. Evidence to date, however, has shown that WtoFE is indirectly linked to family life satisfaction in dual-earner couples [35], and to adolescents’ well-being [43] and SWFoL [38]. Parenting behaviors have also been shown to mediate between the couple’s marital satisfaction and their children’s well-being [57]. Based on these findings, we propose that WtoFE and domain satisfaction are related via positive interactions between family members, such as positive monitoring practices exerted by parents and the adolescent’s perception of their parents’ monitoring practices. 

Against this background, we argue that WtoFE can improve the individual’s performance and fulfilment in the food domain, that is, improving the FPP that working parents exert on their children may enhance the SWFoL of all family members. To the best of the authors’ knowledge, this relationship has not been assessed in the literature. In addition, most studies on FPP have largely focused on mothers with young children rather than adolescent children, or have focused on mother–child dyads only [13,27], neglecting the father’s role in exerting FPP. The father’s role becomes an increasing point of interest, because evidence indicates that fathers have adopted more childcare roles such as feeding, in general due to the increase in dual-earner parents with children [12,51], and particularly during the COVID-19 pandemic [40,58]. 

**Figure 1:**
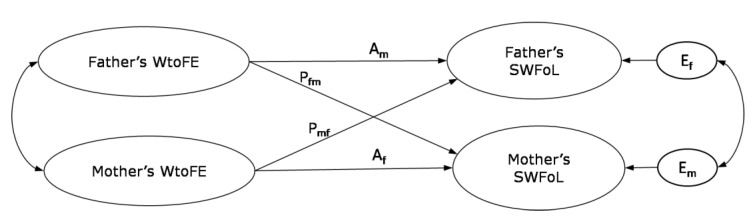
Basic actor–partner interdependence model of work-to-family enrichment (WtoFE) and satisfaction with food-related life (SWFoL). A_m_: actor effect of father’s WtoFE on his own SWFoL; A_f_: actor effect of mother’s WtoFE on her own SWFoL; P_fm_: partner effect of father’s WtoFE on mother’s SWFoL; P_mf_: partner effect of mother’s WtoFE on father’s SWFoL; E_f_ and E_m_: residual errors on SWFoL for the father and mother, respectively.

To fill the above gaps in the literature, following the W-HR model [37], and using the Actor–Partner Interdependence Model (APIM, [59]), the influence of both parents’ WtoFE on their own monitoring practices and SWFoL, as well as on their adolescent’s perception of their parent’s monitoring and SWFoL, were evaluated in parallel. In the APIM (Figure 1), actor effects are the outcomes predicted by the individual’s own characteristics, while partner effects are outcomes from one member of the dyad which are predicted by the characteristics of the other member [59]. The aims of this study were to explore the actor and partner effects between work-to-family enrichment, parents’ monitoring practices, the adolescent’s perception of their parents’ monitoring practices, and the three family members’ satisfaction with food-related life, in different-sex dual-earner parents with adolescent children; and to explore whether parents’ monitoring or adolescent’s perception of their parents’ monitoring have mediating roles between work-to-family enrichment and satisfaction with food-related life. 

To this end, the following hypotheses were posited:

**H1.**  *Work-to-family enrichment is positively associated with satisfaction with food-related life for each parent (actor effects).*

**H2.**  *Work-to-family enrichment of one parent is positively associated with satisfaction with food-related life of (a) the other parent, and (b) of the adolescent (partner effects).*

**H3.**  *Work-to-family enrichment is positively associated with the monitoring practices exerted by each parent (actor effects).*

**H4.**  *Work-to-family enrichment of one parent is positively associated with the monitoring practices exerted by (a) the other parent and (b) the adolescent’s perception regarding their parents’ monitoring practices (partner effects).*

**H5.**  *The monitoring practices exerted by parents and the adolescent’s perception regarding their parents’ monitoring practices are positively associated with their own satisfaction with food-related life for fathers, mothers, and adolescents (actor effects).*

**H6.**  *The monitoring practices exerted by one parent are positively associated with the satisfaction with food-related life of the other parent (partner effects).*

**H7.**  *The adolescent’s perception regarding their parents’ monitoring practices is positively associated with their parents’ satisfaction with food-related (partner effects).*

**H8.**  *Monitoring practices have a mediating role between both parents’ work-to-family enrichment and satisfaction with food-related life for the three family members (actor and partner effects).*

This study was conducted in a Latin American country, Chile. In this regional context, women are expected to be mainly responsible for feeding children and for exerting FPP [16]. We, thus, expected that associations between WtoFE, monitoring practices and SWFoL would show different patterns according to the parent’s gender. However, because the burden of new demands brought on by lockdown measures has been significantly larger for women [5], we expected that fathers would also have a significant involvement in FPP.

## 2. Materials and Methods

### 2.1. Sample and Procedure

The sample was non-probabilistic and comprised 430 dual-earner families from Rancagua, Chile. Inclusion criteria were that these families were composed of a mother and father (married or cohabiting), and at least one adolescent between 10 and 16 years old. Families were recruited via schools serving diverse socioeconomical backgrounds in Rancagua, and answered an online questionnaire, one for each family member (mother, father, and adolescent). Table 1 displays the sociodemographic characteristics of the sample.

After the COVID-19 pandemic was declared in March 2020, lockdown measures were enforced in Rancagua in June and July 2020, and many workers from diverse fields and industries began working from home. Hence, initial contact with families was conducted by phone, via the schools. Trained interviewers invited parents to participate in the study and provided information about the study’s topic and objectives, and confidentiality of the data. Families who agreed to participate received the links to the surveys via e-mail. Data were collected between March and July 2020. 

The first page of the online questionnaire displayed an informed consent form for mothers and fathers, and an assent form for adolescents. All three family members agreed to participate in the study by clicking a box at the bottom of these forms. Each family member registered their responses in the QuestionPro platform (QuestionPro Inc) in a separate questionnaire. After families submitted their questionnaires, they received USD 15 via bank transfer for their participation. 

A pilot test was conducted for this study with fifty families from a different city in Chile. The recruitment method and data collection procedure described above were followed. The results of the pilot test showed that no changes were required for the questionnaire (see Appendix A), nor for the data collection procedure. This study was part of a larger research study on the relations between work, family, and food-related life in Chilean families. This study was approved by the Ethics Committee of the Universidad de La Frontera. 

### 2.2. Measures

The following scale was answered only by mothers and fathers.

Work to Family Enrichment was measured by three items from the Nijmegen’s Work–Home Interaction Survey (SWING; [8,9]). The three items were “You come home cheerfully after a successful day at work, positively affecting the atmosphere at home?”, “You fulfil your domestic obligations better because of the things you have learned at your job”, and “You manage your time at home more efficiently as a result of the way you do your job”. The items were answered using a five-point scale (1: never; 5: very often). This measure was used in its Spanish validated version, which has shown good levels of internal consistency in previous studies in Chile [38,40]. In this study, the standardized factor loadings of this measure were statistically significant (*p* < 0.001) and ranged from 0.577 to 0.807 for mothers, and from 0.742 to 0.809 for fathers. The average variance extracted (AVE) values were higher than 0.50: for mothers = 0.52, and for fathers = 0.59. The Omega coefficient to test internal reliability was acceptable, with values of 0.76 for mothers, and 0.81 for fathers.

The following instruments were answered by the three family members.

Monitoring Practices were measured by four items from the Comprehensive Feeding Practices Questionnaire (CFPQ). The CFPQ [26] is a 12-factor questionnaire that measures twelve different food-related parenting practices, including the monitoring factor. Mothers and fathers answered the validated adapted version, for parents of adolescents, of the monitoring factor [18], that measures the frequency by which parents keep track of a child’s intake of unhealthy foods (i.e., 1. “How much do you keep track of the sweets (ice cream, cake, pastries, chocolates, etc.) that you child eats?”, 2. “How much do you keep track of the snack food (potato chips, Doritos, cheese puffs) that your child eats?”, 3. “How much do you keep track of the high-fat foods that your child eats?”, 4. “How much do you keep track of the sugary drinks that your child drinks ?”). The adolescents answered the validated version of the monitoring factor adapted to be answered by adolescents [15], to assess “the adolescents’ perception regarding the frequency with which parents monitor the consumption of unhealthy foods” (i.e., 1. “How often does this caregiver keep track of the quantity of sweets (or ice cream, cakes, pies, chocolates, candies) that you eat?”, 2. “How often does this caregiver keep track of the quantity of industrialized snacks (potato chips, munchies, cheese pastries, etc.) that you eat?”, 3. “How often does this caregiver keep track of the quantity of fatty foods (hamburgers, snacks, mayonnaise, etc.) that you eat?”, 4. “How often does s/he keep track of the quantity of sweet drinks (soda/soft drinks, juices) that you drink?”). In this study, the Spanish-adapted versions of this measure were used [60]. The authors adapted the four items so as to be equivalent for parents and their adolescents. In the adolescent’s version, the words “this caregiver” were replaced by “your parents”. For the parents’ and adolescent’s versions, the examples of foods were the same and included only food available in the Chilean market. Foods for item 1 were ice cream, cake, pastries and chocolates; for item 2 were potato chips and Doritos; for item 3 were hamburgers and mayonnaise, while for item 4 were soft drinks and juices. The three family members answered each item using a 5-point Likert scale (1: never, 5: always). Melbye and Hansen [19] reported a Cronbach’s alpha of 0.80 for the monitoring dimension of the adapted CFPQ in a sample of parents of adolescents in Norway. Piccoli et al. [15] reported a Cronbach’s alpha of 0.85 for the monitoring dimension of the adapted CFPQ in a sample of adolescents in Brazil. Monitoring scores were obtained by summing the scores from the four items, with higher scores representing higher monitoring in parents and a higher perception of their parents’ monitoring in adolescents. In this study, the standardized factor loadings of the monitoring factor ranged from 0.850 to 0.962 for mothers, from 0.919 to 0.985 for fathers, and from 0.833 to 0.982 for adolescents, all statistically significant (*p* < 0.001). The AVE values were higher than 0.50 (AVE mothers = 0.84, fathers = 0.90, adolescents = 0.85). The monitoring factor showed good internal reliability, the Omega coefficient was 0.95 for mothers, 0.86 for fathers, and 0.96 for adolescents.

Satisfaction with Food-related Life (SWFoL). The SWFoL [41] is a five-item scale that evaluates an individual’s assessment of their food and eating habits (e.g., “Food and meals are positive elements”). Response scale is a six-point Likert scale (1: completely disagree; 6: completely agree). Item responses are summed to obtain the overall scores, with higher scores representing higher SWFoL. The Spanish version of the SWFoL was used [61], which has been validated and tested in samples of adults, adolescents and dual-earner parents in Chile (e.g., [16,21,28,29,44,45,46]. In this study, the standardized factor loadings of the SWFoL scale were statistically significant (*p* < 0.001) and ranged from 0.634 to 0.905 for mothers, from 0.619 to 0.843 for fathers, and from 0.634 to 0.831 for adolescents. The AVE values were higher, at 0.63 for mothers, 0.61 for fathers, and 0.57 for adolescents. The Omega coefficient indicated good internal reliability, with values of 0.89 for mothers, 0.86 for fathers and 0.87 for adolescents.

The three family members reported their age, and adolescents reported their gender. Parents reported their type of employment and number of weekly working hours. Mothers reported the number of family members, number of children, number of days per week in which the family ate a meal together (breakfast, lunch, supper and dinner); the number of days per week that the family consumed homemade food, ready-to-eat food, ordered food at home, or ate at restaurants or fast-food outlets; and the number of daily hours that they, their male partner and/or another person spent cooking during the week and on weekends. Mothers also reported their own approximate weight and height, as well as those from their partner and children, to determine body mass index (BMI, kg/m^2^). Lastly, mothers reported the person who decided on, and purchased, food for the home. Information on household income and size was used to determine the family socioeconomic status (SES, [62]).

### 2.3. Data Analysis

SPSS v.23 was used to conduct descriptive analysis. Mplus 8.4 was used to test the actor–partner interdependence model (APIM) with distinguishable dyads via structural equation modelling (SEM, [59]). In the APIM, associations between variables for an individual are termed “actor effects”, whereas associations between variables from one individual to another (parent-to-parent, or parent-to-child) are “partner effects”. Members of either dyad (mother–father, parent–child) are both an actor and a partner in the analysis. The actor and partner effects tested were both parents’ work-to-family enrichment (WtoFE) and monitoring practices from both parents, the adolescent’s perception regarding their parents’ monitoring practices, and the three family members’ satisfaction with food-related life (SWFoL). 

The APIM allows controlling for effects. First, one parent’s effect on the other in terms of WtoFE was controlled by specifying a correlation between this variable in each parent. Kenny et al. [59] suggested controlling other sources of interdependence between partners by specifying correlations between the residual errors of the dependent variable—in this case, the SWFoL of the three family members. Other variables with direct effects on the dependent variables of three family members (monitoring practices for parents, the adolescent’s perception regarding their parents’ monitoring practices and SWFoL) were controlled. These controlled effects were those of parents’ and adolescent’s age, parents’ type of employment and working hours, the family SES, number of children and times of family suppers per week. 

For the SEM, the structural model parameters were estimated using robust unweighted least squares (ULSMV). As items were on an ordinal scale, the analysis was conducted using the polychoric correlation matrix. The model fit of the data was established following these values: the Tucker–Lewis index (TLI) and the comparative fit index (CFI), which indicate a good fit with values above 0.95; and the root mean square error of approximation (RMSEA), which indicates a good fit when values are below 0.06 [63]. To test the mediating role of monitoring practices, a SEM was conducted through a bias-corrected (BC) bootstrap confidence interval using 1000 samples [64]. A mediating role is identified when BC confidence intervals do not include zero. 

## 3. Results

### 3.1. Sample Description

Table 1 shows the sociodemographic characteristics of the sample, which encompassed 430 families of mother, father and adolescent (53.7% female). Average ages were 38.5 years for mothers, 42.3 for fathers, and 13.0 for adolescents. Average age difference between mothers and fathers was significant (*p* < 0.001). On average, families had four members and two children, and most of these families belonged to a middle SES.

The exploration of food habits showed that family members had breakfast, lunch, and supper together more than three days per week, and frequently consumed homemade food. Mothers and fathers were both the main responsible persons for food purchasing and decision-making in the home, with mothers in the second place. Mothers also spent more hours per day cooking during the week and on weekends than their male partners and other persons (*p* < 0.001), and fathers cooked more hours per day in the same periods than other persons. The category “Other persons” included grandparents, adult children and domestic service. Most mothers and fathers were employees, and a greater proportion of fathers were employees than mothers (*p* < 0.001). A greater proportion of fathers also worked full time (45 h per week in Chile, *p* > 0.001), than mothers. 

According to the norms of the World Health Organization (WHO), 28.4% of the mothers had a body mass index in the normal range (BMI: 18.5–24.9), 44.2% were overweight (BMI: 25.0–29.9) and 27.9% were obese (BMI ≥ 30), whereas 16.3% of the fathers had a body mass index in the normal range, 57.0% were overweight, and 26.7% were obese. According to the reference pattern of the WHO [65] and the Technical Norm of Nutritional Evaluation of children from 5 to 19 years old, of the Ministry of Health of Chile [66], 0.5% of adolescents in this sample had a body mass index that denoted thinness (≤−1 to −1.9 SD), 27.9% were in the normal range (+0.9 to −0.9 SD), 44.2% were overweight (≥+1 to +1.9 SD), and 27.4% were obese (≥+2 SD).

Table 2 shows the average score and correlations for work-to-family enrichment (WtoFE), monitoring, and satisfaction with food-related life (SWFoL). Most of the correlations were significant and in the expected directions, except the correlation between mother’s WtoFE and adolescent’s perception regarding their parents’ monitoring practices, between father’s WtoFE and mother’s SWFoL, and between adolescent’s perception regarding their parents’ monitoring practices and mother’s SWFoL. Mothers and fathers did not differ in the average scores for WtoFE (*t* = −0.107, *p* = 0.915). Mothers scored significantly higher than fathers and adolescents in monitoring (*F* = 43.812, *p* < 0.001), while adolescents scored significantly higher than fathers. Adolescents scored significantly higher than their mothers in SWFoL (*F* = 26.463, *p* < 0.001), while fathers did not differ from mothers and adolescents.

### 3.2. APIM Results: Testing Actor–Partner Hypotheses

**Figure 2:**
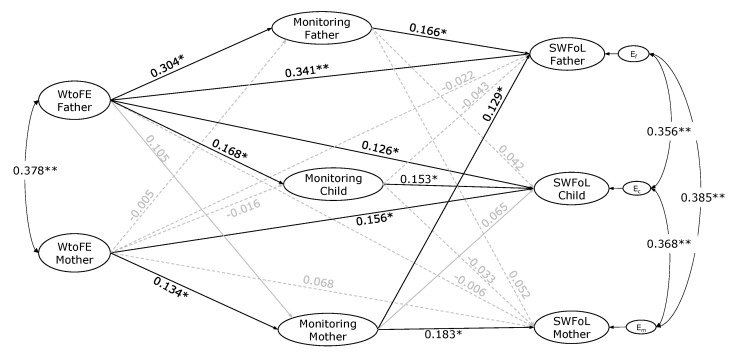
Actor–partner interdependence model of the effect of parents’ work-to-family enrichment (WtoFE) on the three family members’ monitoring (the perception regarding parents’ monitoring practices in adolescents) and satisfaction with food-related life (SWFoL) in dual-earner parents with adolescent children. E_f_, E_c_ and E_m_: residual errors on SWFoL for the fathers, mothers and their adolescent children, respectively. * *p* < 0.05, ** *p* < 0.01. The control for the effects of both members of the couple’s age, type of employment and their number of working hours as well as the family SES, the number of children and the number of supper times in which all the family members ate together during a week on the dependent variables of the three family members (monitoring and SWFoL), are not shown in the path diagram.

The results from the estimation of the structural model are shown in Figure 2. The model that assessed the APIM association between the mother’s and father’s work-to-family enrichment (WtoFE), both parents’ monitoring, their adolescent children’s perception regarding their parents’ monitoring practices, and the three family members’ satisfaction with food-related life (SWFoL) had an acceptable fit with the data (CFI = 0.941; TLI = 0.931; RMSEA = 0.025). A significant correlation (covariance) was found between WtoFE of both parents (*r* = 0.378, *p* < 0.001). Significant correlations were also found between the residual errors of the mother’s and the father’s SWFoL (*r* = 0.385, *p* < 0.001), between the mother’s and the adolescent’s SWFoL (*r* = 0.368, *p* < 0.001), and between the father’s and the adolescent’s SWFoL (*r* = 0.356, *p* < 0.001).

H1 stated that work-to-family enrichment is positively associated with satisfaction with food-related life for each parent. As shown in Figure 2, the direct path coefficients (standardized) indicated that the father’s WtoFE was positively associated with his own SWFoL (*γ* = 0.341, *p* < 0.001). By contrast, the mother’s WtoFE was not directly associated with her own SWFoL (*γ* = 0.068, *p* = 0.216). These findings supported H1 only for fathers; however, there may still be an indirect effect for mothers, mediated by monitoring practices, which we will address below.

H2 examined partner effects, stating that work-to-family enrichment of one parent is positively associated with the SWFoL of the other parent (H2a) and that of the adolescent (H2b). The father’s WtoFE was not directly associated with the mother’s SWFoL (*γ* = −0.006, *p* = 0.922). Similarly, the mother’s WtoFE was not directly associated with the father’s SWFoL (*γ* = −0.022, *p* = 0.660). By contrast, the father’s (*γ* = 0.126, *p* = 0.042) and mother’s (γ = 0.156, *p* = 0.006) WtoFE were directly positively associated with the adolescent’s SWFoL. H2b was, therefore, supported for both parents. H2a could still be supported by indirect effects, which we will address below.

H3 tested actor effects, stating that work-to-family enrichment is positively associated with the monitoring practices exerted by each parent. The path coefficients indicated that both the father’s (γ = 0.304, *p* = 0.002) and the mother’s (*γ* = 0.134, *p* = 0.036) WtoFE was positively associated with their own monitoring practices. These findings supported H3.

H4 tested partner effects, stating that work-to-family enrichment of one parent is positively associated with the monitoring practices of the other parent (H4a) and the adolescent’s perception regarding their parents’ monitoring practices (H4b). Results showed that the father’s WtoFE was not significantly associated with the mother’s monitoring (*γ* = 0.105, *p* = 0.090), nor was the mother’s WtoFE significantly associated with the father’s monitoring (*γ* = −0.005, *p* = 0.927). While the father’s WtoFE (*γ* = 0.168, *p* = 0.014) was positively associated with the adolescent’s perception regarding their parents’ monitoring practices, the mother’s WtoFE was not (*γ* = −0.016, *p* = 0.774). These findings did not support H4a for parents, whereas they partially supported H4b for adolescents.

H5 tested actor effects for the three family members, namely, that the monitoring practices exerted by parents, and the adolescent’s perception regarding their parents’ monitoring practices, are positively associated with their own SWFoL for fathers, mothers, and adolescents. The path coefficients indicated that the monitoring practices exerted by fathers (*γ* = 0.166, *p* = 0.018) and mothers (*γ* = 0.183, *p* = 0.003) were positively associated with their own SWFoL. Similarly, the adolescent’s perception regarding their parents’ monitoring practices (*γ* = 0.153, *p* = 0.033) was positively associated with their own SWFoL. These findings supported H5.

H6 tested partner effects, stating that the monitoring practices exerted by one parent are positively associated with the SWFoL of the other parent. The father’s monitoring was not significantly associated with the mother’s SWFoL (*γ* = 0.052, *p* = 0.389). By contrast, the mother’s monitoring was positively associated with the father’s SWFoL (*γ* = 0.129, *p* = 0.043). These findings partially supported H6.

H7 stated that the adolescent’s perception regarding their parents’ monitoring practices is positively associated with their parents’ satisfaction with food-related life. However, the findings showed that the adolescent’s perception regarding their parents’ monitoring practices was not significantly associated with the father’s SWFoL (*γ* = 0.043, *p* = 0.492) nor with the mother’s SWFoL (*γ* = −0.033, *p* = 0.607), and, thus, H7 was not supported. 

Most of the control variables did not affect the model significantly (Table 3). The number of family supper times per week positively affected the mother’s (*γ* = 0.111, *p* < 0.05), the father’s (*γ* = 0.133, *p* < 0.01) and the adolescent’s (*γ* = 0.111, *p* < 0.05) SWFoL, while the family SES positively affected the mother’s SWFoL (*γ* = 0.143, *p* < 0.01); that is, mothers belonging to a higher SES experienced a higher level of satisfaction with food-related life than those of lower SES. The adolescent’s age negatively affected the mother’s (*γ* = −0.174, *p* < 0.01) and the father’s (*γ* = 0.142, *p* < 0.05) monitoring as well as the adolescent’s perception regarding their parents’ monitoring practices (*γ* = −0.282, *p* < 0.001). The mother’s type of employment positively affected her own monitoring (*γ* = 0.111, *p* < 0.05); that is, self-employed mothers had higher levels of monitoring than employed mothers. The father’s working hours positively affected their own monitoring, meaning that fathers working less than 45 h per week had higher levels of monitoring than fathers working 45 h per week.

### 3.3. Testing Mediating Roles of Monitoring

The last hypothesis of this study tested the mediating role of both parents’ monitoring practices between their own work-to-family enrichment and satisfaction with food related life, as well as the mediating role of the adolescent’s perception of their parents’ monitoring practices between each parent’s work-to-family enrichment and the adolescent’s satisfaction with food-related life (H8). The role of the father’s monitoring practices as mediator in the relationship between his own WtoFE and SWFoL was supported by a significant indirect effect obtained with the bootstrapping confidence interval procedure (standardized indirect effect = 0.054, 95% CI (010, 0.097)), as the confidence intervals did not include zero (Table 4). No other indirect effects of monitoring were found, as the confidence intervals did include zero (Table 4). These findings partially supported the mediating role of monitoring between parents’ work-to-family enrichment and their own SWFoL, while they did not support the mediating role of the adolescent’s perception regarding their parents’ monitoring practices. The mediation analysis, thus, provided additional support for H1 only for fathers but not for mothers, and did not provide support for H2a. H2a must, therefore, be rejected.

## 4. Discussion

Parents’ eating behaviors and the overall family food environment have a strong influence on the development of their children’s eating behaviors [13,15,27,46]. Food parenting practices (FPP) are part of this environment and can play a role in the development of overweight and obesity in children and adolescents [13]. While there is increasing evidence showing that numerous family-related variables influence the parents’ use of different FPP (e.g., [21,23,24,27,28,29]), less is known about the influence of parents’ work-related factors on the use of FPP and their effects [38,40]. This study provides new insights regarding work-related factors that may influence parents’ FPP by showing that work-to-family enrichment (WtoFE), i.e., drawing on resources acquired in the workplace to perform tasks at home, can promote positive outcomes in the food domain. The outcomes examined in this study were parental food monitoring practices, the adolescent’s perception of these practices, and satisfaction with food-related life (SWFoL). These variables are relevant, as research shows they are associated with healthier eating habits and higher life satisfaction and well-being in both adults and adolescents [16,19,21,22,23,25,29,67,68]. Using the actor–partner interdependence model (APIM), our results showed that higher WtoFE can enhance the parents’ monitoring practices, the adolescent child’s perception of their parents’ monitoring and SWFoL, in dual-earner parents and their adolescent children, although different patterns emerged in these relationships according to the parent’s gender. 

In agreement with the W-HR model [37], the positive relationship between WtoFE and SWFoL for fathers suggests that resources acquired by fathers in the work domain (i.e., mood, efficiency, time) can be transferred to the food domain, facilitating a positive performance of food-related tasks, such as purchasing healthy foods and preparing healthy meals. These latter tasks have been linked to higher levels of SWFoL in adult samples [20,21,46]. This result supports that WtoFE increases workers’ well-being in different life domains [35,42], including the food domain [38], and that resources provided by a positive work–home balance can enhance the male’s SWFoL in dual-earner couples [44]. However, this evidence did not seem to uphold for the mothers in this sample, as results showed that mothers’ resources acquired from work did not improve their own SWFoL. This finding was contrary to expectations and previous studies showing that higher work–home balance and WtoFE enhances the woman’s SWFoL in dual-earner couples [38,44]. Moreover, this distinction was found despite fathers and mothers having similar WtoFE scores. 

However, the aforementioned studies were conducted before the COVID-19 pandemic [38,44]. The lack of relationship between WtoFE and SWFoL for mothers may be reflecting the higher burden of the new demands brought on by the pandemic for women [5]. The COVID-19 pandemic has multiplied domestic demands for women regardless of their employment status, and it has forced them to merge their work and family spaces [1]. Research during the pandemic in developed countries showed that women remained responsible for household chores, such as cleaning and doing the laundry [58]. In this context, it can be hypothesized that resources that mothers have acquired from work have been used to face other domestic tasks rather than food-related tasks during the pandemic, and, thus, WtoFE has not resulted in a higher SWFoL for them. 

Contrary to expectations based on the W-HR model [37], there was no association between one parent’s WtoFE and the other parent’s SWFoL. These findings also contradicted previous studies supporting WtoFE crossover effects between couples [35,36,42], and those showing that resources provided by a high balance between work and home positively influenced both members of the couple’s SWFoL in dual-earner families [44]. Regarding the positive relationships between each parent’s WtoFE and their adolescent child’s SWFoL, our results were in line with previous research showing that the mother’s and father’s higher balance between their work and home had a positive influence on their adolescent child’s SWFoL [45]. The scant literature testing WtoFE crossover from parents to their adolescent child’s well-being has shown that the mother’s WtoFE indirectly influenced her adolescent child’s well-being through the quality of the mother–child relationship [43], and that both parents’ WtoFE indirectly influenced their adolescent child’s SWFoL via the adolescent’s positive perception of the atmosphere of family meals [38]. However, our results are novel because they show that both parents’ WtoFE directly influences their adolescent child’s SWFoL. One possible explanation for this effect was that the WtoFE allowed parents to prepare healthy meals or promote having more frequent family meals, two factors that have been associated with an adolescent’s SWFoL [20,21]. In this line, in the present study, the frequency of family meals was higher than in studies conducted in Chile before the pandemic [21,38,46].

Interestingly, while the father’s WtoFE was positively associated with their own and their adolescent children’s SWFoL, the mother’s WtoFE was only positively associated with their adolescent child’s SWFoL. These results suggest that mothers in this sample were also able to transfer resources gained in the work domain to the food domain, but it may be the case that these resources were allocated to the additional child-rearing tasks associated with the pandemic conditions [1]. Thus, mothers were able to improve their adolescent children’s SWFoL, but not their own. Although further research is needed to better understand these relationships, one possible explanation may be related to the traditional gender-based demands and expectations in the Latin American context, where feeding children is still predominantly female labor [16]. Therefore, it is possible that mothers used their work resources in food-related activities that positively influenced their children’s SWFoL, but with no effect on their own SWFoL. Future research should assess the relationships between a parent’s WtoFE and the family members’ SWFoL, as well as whether working mothers have gained enough resources from the work domain to influence their own and their family members’ SWFoL beyond the pandemic.

Correspondingly, in agreement with the W-H R model, our results showed that work-to-family enrichment was positively associated with the monitoring practices exerted by mothers and fathers. These findings indicated that WtoFE may improve the parents’ performance in the food domain and, thus, improve their monitoring practices, regardless of the parent’s gender. It should be noted, however, that the path coefficients between WtoFE and monitoring were low for mothers and medium for fathers, while mothers had an average monitoring score significantly higher than fathers. This may be related to the increased involvement of fathers in their children’s care [40,58] during the COVID-19 pandemic, such that fathers had more need for the transfer of resources obtained at work. At the same time, it could be suggested that mothers may have acquired resources from other sources besides work (e.g., family support) that allowed them to achieve a higher level of monitoring, in comparison with fathers.

Contrary to expectations, our results showed that work resources acquired by one parent did not cross over to the other parent’s monitoring practices. We also found that only the father’s work resources crossed over to their adolescent child’s perception of their parents’ monitoring practices. Regarding the lack of partner effects from the mother’s WtoFE to the father’s monitoring and to their adolescent child’s perception of monitoring practices, one possible explanation may be the low strength relationship between WtoFE and monitoring in mothers, because the literature shows that if actor effects are low or non-significant, it is likely that partner effects do not exist [69]. Therefore, only the results for fathers and their adolescent children were consistent with the W-HR model [37], which states that parents may use resources acquired in the work domain, through WtoFE, to promote better parent–child interactions [43,48], which, thus, may positively influence the adolescent’s perception of their parents’ monitoring practices.

This latter result is also in line with previous studies showing that parents with more resources provided by a high balance between work and home demands are more involved in their children’s eating behavior [51] and prepare healthier meals for their family members [52,53]. However, as previously noted, mothers and fathers did not differ in their WtoFE scores. Therefore, the lack of relationship between the mother’s WtoFE and their adolescent’s perception of their parents’ monitoring practices, as well as the positive relationship in the case of fathers, may be associated with the high burden experienced by working women during the pandemic. That is, mothers may have turned to other home-related tasks rather than monitoring their adolescent children [5], while fathers increased their involvement in childcare and food-related tasks during this period [40,58]. Both situations may have influenced adolescents’ perception of their parents’ monitoring practices. For instance, adolescents may have noticed a higher participation of fathers, alone or along with mothers, in food decision-making and purchasing during the pandemic, as was observed in the present study, in comparison with previous studies in Chile [46]. By contrast and considering the traditional family structure prevailing in Latin American countries [16], it is also possible that adolescents are accustomed to the monitoring practices exercised by their mothers and have not noticed changes associated with the resources acquired by their mothers from the work domain during the pandemic. Nevertheless, further research should corroborate these results beyond the COVID-19 pandemic.

Regarding both parents’ positive association between their own monitoring and SWFoL, this result may have reflected the parents’ own eating behaviors [27], which can result in higher levels of SWFoL [20,21,46]. However, it was also possible that the positive association between both parents’ monitoring and their own SWFoL may have been related to the possibility of improving their children’s diet quality, as a previous study reported that parents’ SWFoL is also related to their family members’ healthy eating behaviors [20]. In addition, our results not only showed that the mother’s SWFoL may be positively influenced by their own exertion of positive FPP, but that this association was also found in fathers. In part, this finding contradicts a previous study in Chile in which FPP exerted by fathers negatively influenced their own life satisfaction [16]. Although further research is needed regarding the father’s FPP and their own and their children’s outcomes [13,27], this result may be specific to the pandemic context, reflecting the increased involvement of fathers in their children’s eating habits during this crisis [58]. Therefore, future research should assess if the positive association between fathers’ monitoring and SWFoL remains beyond the pandemic, while a possible mediating role of SWFoL between fathers’ FFP and life satisfaction may also be tested. Regarding the positive association between monitoring and SWFoL in adolescent children, this finding was in line with those reported in samples with adolescent children that associate parents’ monitoring practices with positive diet outcomes in their children [19,22,23,25], which, in turn, have been associated with higher SWFoL in adolescents [20,21,46]. The positive association found here between the adolescent perception regarding their parents’ monitoring practices and SWFoL may indicate that adolescents appreciate their parents’ concern to promote healthy food choices to prevent health issues such as those related to overweight and obesity [25].

Regarding partner effects between both parents’ monitoring and SWFoL, only the mother’s monitoring practices were related to the father’s SWFoL, but not vice versa. One possible explanation for these results may be associated with the higher monitoring scores for mothers in comparison with fathers, so it is feasible that monitoring practices exerted by fathers were not enough to positively influence the mothers’ SWFoL. Indeed, although both mother’s and father’s SWFoL were associated with providing a healthy diet to their family members, this association was more relevant for mothers [20]. These results, however, may also be related to the different socialization practices and social roles for women and men. In Latin America, including Chilean different-sex dual-earner families, men are still considered as the main provider for the household [70] while mothers are expected to feed the family [16]. Therefore, it is possible to hypothesize that the positive association between the mother’s monitoring and the father’s SWFoL was due to mothers fulfilling their traditional gender role, while the lack of relationship between father’s monitoring and mother’s SWFoL may be reflecting that mothers do not account for the participation of fathers in children’s monitoring, because this task is not associated with the traditional role of the father within the family. Therefore, further research is needed in countries where the relationship between different-sex couples is more egalitarian. Regardless of the above, our results provide new insights regarding the relationship between FPP and SWFoL, showing that FPP may not only positively influence the SWFoL of the parent who exerts the FPP [16,21,28], but also that of their partner through crossover. Future research should also evaluate possible crossovers to other indicators of well-being, such as emotional well-being, mental health and life satisfaction.

No partner effects from the adolescent child’s perception of their parents’ monitoring practices to their parent’s SWFoL were found. Partner effects were expected on the grounds that the parents’ SWFoL were also related to their family members’ healthy eating behaviors [20]. Instead, this finding might suggest that when parents evaluate their food-related life, they do not include their adolescent child’s perception of their monitoring practices. However, it is also possible that adolescents do not express their perceptions regarding the FPP that their parents exert on them.

Lastly, contrary to what was hypothesized, monitoring showed a mediating role between WtoFE and SWFoL only for fathers. These findings, thus, indicated that WtoFE and SWFoL were associated indirectly via monitoring for fathers, contributing to previous studies suggesting the existence of intra-individual mediators between WtoFE and well-being outcomes in workers [35]. Although further research is needed to explain the lack of a mediating role of monitoring in mothers, this null relationship may be related to the low strength relationship between their own WtoFE and monitoring. On the other hand, the lack of mediating role for the adolescent’s perception of their parents’ monitoring practices contradicted previous findings showing that an adolescent’s perception of the quality of their relationship, perception of the atmosphere of family meals, or parenting behaviors serves a mediating role between parents’ variables and children’s well-being [38,43,57]. This lack of a mediating role of the adolescents’ perception of their parents’ monitoring may be similar to that obtained in mothers. 

The limitations of this study are acknowledged. First, the APIM language refers to actor and partner effects, but this is a cross-sectional study and causal relationships cannot be established. Second, the sample was self-selected. We took measures to ensure that this sample was representative of the distribution by socioeconomic status in Chile [62], but these families had, overall, more members than the average Chilean family [71]. Another limitation was that responses were self-reported, which may have allowed participants to respond based on social desirability regarding their work, monitoring practices, and their food-related life. Lastly, this research was designed and started before the COVID-19 pandemic, and, thus, conditions related to this context (e.g., working from home or commuting during lockdown) were not explored. These limitations could be overcome by future research, by considering longitudinal designs and probabilistic samples. Moreover, cross-cultural comparisons will allow for accounting for varying degrees of gender equality, and of restrictions and consequences of the COVID-19 pandemic in diverse populations. 

## 5. Conclusions

This study examined intra-individual and inter-individual effects for the relationships between parents’ work-to-family enrichment (WtoFE) and monitoring practices, the adolescent’s perception of their parents’ monitoring practices, and the three family members’ satisfaction with food-related life, in different-sex dual-earner parents with adolescent children. The results showed that resources acquired by both parents from the work domain, through WtoFE, enhanced their own monitoring practices, which, in turn, improved their own SWFoL. Similarly, both parents’ WtoFE enhanced their adolescent child’s SWFoL, while the adolescent’s perception of their parents’ monitoring practices also improved their own SWFoL. However, different gender patterns emerged among parents regarding the influence of WtoFE and monitoring, on their own and their adolescent child’s SWFoL. Results showed a positive association between WtoFE and SWFoL, directly and through monitoring only in fathers, while the father’s SWFoL was also positively associated with the mother’s monitoring, but not vice versa. Lastly, only the father’s WtoFE was positively associated with the adolescent’s perception of their parents’ monitoring practices. These findings show that both parents’ WtoFE contributed to improving their own monitoring and the adolescent’s SWFoL during the first months of the COVID-19 pandemic. These results also underscore the significant role of a father’s WtoFE in improving their adolescent’s perceptions of parental monitoring practices. 

The findings from this study have practical implications. Public health campaigns should provide easily accessible information on healthy and unhealthy food intake, both aimed at adolescent and adult populations. These campaigns should encourage parents to use this information to monitor their adolescents’ intake of unhealthy foods, and adolescents to also monitor their own food intake outside the home (i.e., at school). For instance, nutritional education campaigns aimed to parents can describe positive FPP and how they can be exerted, while emphasizing not only the nutritional positive outcomes, but also the positive outcomes for the individual and family health and well-being. Our results also underscore the need for organizations and policymakers to create conditions that promote work-to-family enrichment (see, e.g., [35]). Public and private organizations are advised to enforce politics and interventions (e.g., a family-friendly cultural organization, organizational health campaigns) that provide workers with personal resources (e.g., time, skills, knowledge), to encourage them to invest those resources in their own food-related behaviors, and to improve their family’s nutritional and social (e.g., family meals) aspects of food consumption. Special attention is required for the needs of working mothers, such as flexible schedules and a supportive work–home organization culture. 

These findings also pose research implications. The results show that both parents’ WtoFE improved their monitoring practices, which, in turn, improved their own SWFoL as well as their adolescent child’s SWFoL. Therefore, further research should assess these relationships including other structured FPP, as well as FPP included in the coercive control and autonomy support or promotion classification by Vaughn et al. [14]. Given that different patterns emerged according to the parent’s gender, future research should corroborate if these differences were associated with the COVID-19 pandemic, or whether they may persist beyond the pandemic. In addition, further research should assess the type of work resources (i.e., time flexibility, skills, support from supervisors and co-workers) that may allow working parents to positively influence their FPP and their own, as well as their children’s, SWFoL, particularly for working mothers during a pandemic or other social crisis or emergencies. Future research should also explore possible moderators of the associations found in this studyor instance, the frequency of family meals, the family SES, the mother’s type of employment, the father’s number of working hours and the adolescent’s age, as control variables significantly affected the monitoring or SWFoL.

## Figures and Tables

**Table 1:** Sample characteristics (*n* = 430).

Characteristic	Total Sample	*p*-Value ^1^
Age (mean (SD)) ^1^		
Mother	39.5 (6.6)	<0.001
Father	42.3 (7.8)	
Adolescent	13.0 (2.0)	
Adolescent’s gender (%)		
Male	46.3	
Female	53.7	
Number of family members (mean (SD))	4.3 (1.0)	
Number of children (mean (SD))	2.2 (0.8)	
Socioeconomic status (%)		
High	3.7	
Middle	83.0	
Low	3.7	
Number of days per week that families ate together (mean (SD))		
Breakfast	3.5 (2.7)	
Lunch	4.9 (2.4)	
Supper	6.0 (1.9)	
Dinner	2.2 (3.1)	
Number of days families ate different types of foods (mean (SD))		
Homemade foods	6.4 (1.3)	
Buy ready-to-eat food	0.4 (1.2)	
Order food at home	0.6 (0.7)	
Eat at restaurants	0.2 (0.5)	
Eat at fast-food outlets	0.3 (0.6)	
Person who decides to buy food (%)		
Mother	44.2	
Father	2.8	
Both parents	50.7	
All (mother, father, and children)	1.9	
Another person	0.5	
Person who purchases the food (%)		
Mother	37.0	
Father	5.3	
Both parents	54.0	
All (mother, father, and children)	3.3	
Another person	0.5	
Number of hours per day spent cooking during the week (mean (SD)) ^2^		
Mother	2.6 (1.3)	<0.001
Father	1.2 (1.3)	
Another person	0.9 (1.5)	
Number of hours per day spent cooking on the weekend (mean (SD)) ^2^		
Mother	3.1 (1.6)	<0.001
Father	1.7 (1.4)	
Another person	0.7 (1.2)	
Type of employment (%) ^3^		
Woman employee	62.8	<0.001
Woman self-employed	37.2	
Man employee	75.3	
Man self-employed	24.7	
Working hours (%) ^3^		
Woman working 45 h per week	44.0	<0.001
Woman working less than 45 h per week	56.0	
Man working 45 h per week	67.2	
Man working less than 45 h per week	32.8	

^1^ Independent sample *t*-test. ^2^ Analysis of variance. ^3^ *p*-value corresponds to the (bilateral) asymptotic significance obtained in Pearson’s Chi-square test.

**Table 2:** Descriptive statistics and correlations for parent’s work-to-family enrichment (WtoFE), both parents’ monitoring practices, their adolescent child’s perception regarding their parents’ monitoring practices (monitoring) and the three family members’ satisfaction with food-related life (SWFoL) in dual-earner parents with adolescent children (*n* = 430).

	M (SD)	Correlations							
		1	2	3	4	5	6	7	8
1. Mother’s WtoFE	10.23 (2.80)	-	0.309 **	0.139 **	0.105 *	0.028	0.097 *	0.104 *	0.176 *
2. Father’s WtoFE	10.25 (2.91)		1	0.128 **	0.278 **	0.131 **	0.068	0.318 **	0.157 **
3. Mother’s monitoring	15.28 (3.96)			1	0.424 **	0.457 **	0.138 **	0.208 **	0.157 **
4. Father’s monitoring	12.96 (5.03)				1	0.475 **	0.100 *	0.286 **	0.196 **
5. Adolescent’s monitoring	14.94 (4.71)					1	0.047	0.203 **	0.204 **
6. Mother’s SWFoL	22.13 (4.52)						1	0.303 **	0.298 **
7. Father’s SWFoL	23.13 (4.30)							1	0.351 **
8. Adolescent’s SWFoL	23.94 (4.35)								1

Data analysis was performed using Pearson’s correlation coefficient. * Correlation is significant at the 0.05 level (2-tailed). ** Correlation is significant at the 0.01 level (2-tailed).

**Table 3:** Standardized effects estimate of control variables on satisfaction with food-related life (SWFaL) and monitoring (the perception regarding their parents’ monitoring practices in adolescents) in dual-earner parents with adolescent children.

	Estimate	*p*-Value
Mother’s age → Mother’s SWFoL	0.042	0.534
Father’s age → Mother’s SWFoL	0.096	0.156
Adolescent’s age → Mother’s SWFoL	0.051	0.341
Number of children → Mother’s SWFoL	0.075	0.160
Mother’s type of employment → Mother’s SWFoL	0.064	0.243
Mother’s working hours → Mother’s SWFoL	0.044	0.430
Father’s type of employment → Mother’s SWFoL	0.025	0.619
Father’s working hours → Mother’s SWFoL	−0.091	0.079
Number of supper per week ate together → Mother’s SWFoL	0.111	0.026 *
Family socioeconomic status → Mother’s SWFoL	0.143	0.004 **
Mother’s age → Father’s SWFoL	0.041	0.464
Father’s age → Father’s SWFoL	−0.072	0.208
Adolescent’s age → Father’s SWFoL	0.080	0.140
Number of children → Father’s SWFoL	−0.077	0.145
Mother’s type of employment → Father’s SWFoL	−0.058	0.270
Mother’s working hours → Father’s SWFoL	0.088	0.109
Father’s type of employment → Father’s SWFoL	0.086	0.084
Father’s working hours → Father’s SWFoL	−0.080	0.119
Number of supper times per week ate together → Father’s SWFoL	0.133	0.005 **
Family socioeconomic status → Father’s SWFoL	−0.041	0.462
Mother’s age → Adolescent’s SWFoL	0.048	0.440
Father’s age → Adolescent’s SWFoL	−0.021	0.728
Adolescent’s age → Adolescent’s SWFoL	−0.032	0.576
Number of children → Adolescent’s SWFoL	0.043	0.423
Mother’s type of employment → Adolescent’s SWFoL	0.061	0.275
Mother’s working hours → Adolescent’s SWFoL	0.049	0.373
Father’s type of employment → Adolescent’s SWFoL	0.073	0.176
Father’s working hours → Adolescent’s SWFoL	−0.095	0.072
Number of supper times per week ate together → Adolescent’s SWFoL	0.127	0.009 **
Family socioeconomic status → Adolescent’s monitoring	0.025	0.623
Mother’s age → Mother’s monitoring	0.019	0.738
Father’s age → Mother’s monitoring	−0.020	0.753
Adolescent’s age → Mother’s monitoring	−0.174	0.003 **
Number of children → Mother’s monitoring	−0.051	0.364
Mother’s type of employment → Mother’s monitoring	0.121	0.041 *
Mother’s working hours → Mother’s monitoring	−0.023	0.692
Father’s type of employment → Mother’s monitoring	−0.052	0.334
Father’s working hours → Mother’s monitoring	0.053	0.327
Number of supper times per week ate together → Mother’s monitoring	−0.004	0.942
Family socioeconomic status → Mother’s monitoring	0.080	0.123
Mother’s age → Father’s monitoring	−0.011	0.854
Father’s age → Father’s monitoring	−0.066	0.310
Adolescent’s age → Father’s monitoring	−0.142	0.023 *
Number of children → Father’s monitoring	0.045	0.384
Mother’s type of employment → Father’s monitoring	0.067	0.239
Mother’s working hours → Father’s monitoring	−0.035	0.532
Father’s type of employment → Father’s monitoring	−0.018	0.731
Father’s working hours → Father’s monitoring	0.168	0.011 *
Number of supper times per week ate together → Father’s monitoring	0.092	0.113
Family socioeconomic status → Father’s monitoring	0.064	0.230
Mother’s age → Adolescent’s monitoring	0.067	0.219
Father’s age → Adolescent’s monitoring	−0.042	0.485
Adolescent’s age → Adolescent’s monitoring	−0.282	0.000 **
Number of children → Adolescent’s monitoring	−0.037	0.485
Mother’s type of employment → Adolescent’s monitoring	0.107	0.056
Mother’s working hours → Adolescent’s monitoring	−0.038	0.477
Father’s type of employment → Adolescent’s monitoring	−0.036	0.477
Father’s working hours → Adolescent’s monitoring	0.094	0.081
Number of supper times per week ate together → Adolescent’s monitoring	0.028	0.588
Family socioeconomic status → Adolescent’s monitoring	−0.059	0.235

Data analysis was performed using structural equation modelling. * *p* < 0.05, ** *p* < 0.01.

**Table 4:** Bias-corrected confidence intervals of specific mediation effects of the three family members’ monitoring (the perception regarding parents’ monitoring practices in adolescents).

Effects	Lower 2.5%	Estimate	Upper 2.5%
From mother’s WtoFE to mother’s SWFoL Specific indirect			
Mother’s SWFoL			
Mother’s monitoring			
Mother’s WtoFE	−0.002	0.037	0.075
From father’s WtoFE to father’s SWFoL Specific indirect			
Father’s SWFoL			
Father’s monitoring			
Father’s WtoFE	0.01	0.054	0.097
From parent’s WtoFE to adolescent’s SWFoL			
Specific indirect			
Adolescent’s SWFoL			
Adolescent’s monitoring			
Mother’s WtoFE	−0.031	−0.004	0.023
Adolescent’s SWFoL			
Adolescent’s monitoring			
Father’s WtoFE	−0.006	0.026	0.057

Data analysis was performed using structural equation modelling through a bias-corrected (BC) bootstrap confidence interval using 1000 samples. WtoFE: work-to-family enrichment. SWFoL: satisfaction with family life.

## Data Availability

Data and materials are available from the corresponding author upon reasonable request.

## Appendix A

### Appendix A.1. Questionnaire for Mothers

1. Please indicate how often you experience the following situations (mark only one response per row):

	Never	Almost never	Frequently	Often	Very often
1. You come home cheerfully after a successful day at work, positively affecting the atmosphere at home.					
2. You fulfil your domestic obligations better because of the things you have learned at your job.					
3. You manage your time at home more efficiently as a result of the way you do your job.					

2. Please indicate how often you experience the following situations (mark only one response per row):

	Never	Almost never	Sometimes	Often	Always
1. How much do you keep track of the sweets (ice cream, cake, pastries, chocolates, etc.) that your child eats?					
2. How much do you keep track of the snack food (potato chips, Doritos, etc.) that your child eats?					
3. How much do you keep track of the high-fat foods (hamburgers, mayonnaise, etc.) that your child eats?					
4. How much do you keep track of the sugary drinks (soft drinks, juices) that your child drinks?					

3. Indicate the degree of agreement or disagreement with the following statements (mark only one answer for each row):

	Completely disagree	Mostly disagree	Slightly disagree	Slightly agree	Mostly agree	Completely agree
1. Food and meals are positive elements.						
2. I am generally pleased with my food.						
3. My life in relation to food and meals is close to ideal.						
4. With regard to food, the conditions of my life are excellent.						
5. Food and meals give me satisfaction in my daily life.						

Number of family members
How many children do you have that live with you?

	0	1	2	3	4	5	6	7
1. How many days per week did your entire family, who live with you at home, eat together for breakfast?								
2. How many days per week did your entire family, who live with you at home, eat together for lunch?								
3. How many days per week did your entire family, who live with you at home, eat together for supper?								
4. How many days per week did your entire family, who live with you at home, eat together for dinner?								

	0	1	2	3	4	5	6	7
1. How many days per week did your entire family, who live with you at home, consume homemade food?								
2. How many days per week did your entire family, who live with you at home, consume ready-to-eat food?								
3. How many days per week did your entire family, who live with you at home, order food at home?								
4. How many days per week did your entire family, who live with you at home, eat at restaurants?								
5. How many days per week did your entire family, who live with you at home, eat at fast-food outlets?								

During the week	How many hours per day do you normally spend cooking for your family?	
	How many hours per day does your partner spend cooking for your family?	
	How many hours per day does another adult spend cooking for your family?	
During the weekend	How many hours per day do you normally spend cooking for your family?	
	How many hours per day does your partner spend cooking for your family?	
	How many hours per day does another adult spend cooking for your family?	
	Who is the other adult? During the week:	
	Who is the other adult? During the weekend:	

Weight (kg) 1. Father	
Weight (kg) 2. Mother	
Weight (kg) 3. Child	
Height (cm) 1. Father	
Height (cm) 2. Mother	
Height (cm) 3. Child	

Who decides on and purchases food for the home?	
1. Mother	
2. Father	
3. Both parents	
4. Both parents and children	
5. Other, who?	

How old are you?

What is your employment type?	
1. Dependent	2. Independent

What is your current workday?	
1. Full time job (45 h per week)	
2. Part-time job (less than 45 h per week)	

Please indicate the total income of your household in an average month, considering the contribution of all its members	

### Appendix A.2. Questionnaire for Fathers

1. Please indicate how often you experience the following situations (mark only one response per row):

	Never	Almost never	Frequently	Often	Very often
1. You come home cheerfully after a successful day at work, positively affecting the atmosphere at home.					
2. You fulfil your domestic obligations better because of the things you have learned at your job.					
3. You manage your time at home more efficiently as a result of the way you do your job.					

2. Please indicate how often you experience the following situations (mark only one response per row):

	Never	Almost never	Sometimes	Often	Always
1. How much do you keep track of the sweets (ice cream, cake, pastries, chocolates, etc.) that your child eats?					
2. How much do you keep track of the snack food (potato chips, Doritos, etc.) that your child eats?					
3. How much do you keep track of the high-fat foods (hamburgers, mayonnaise, etc.) that your child eats?					
4. How much do you keep track of the sugary drinks (soft drinks, juices) that your child drinks?					

3. Indicate the degree of agreement or disagreement with the following statements (mark only one answer for each row):

	Completely disagree	Mostly disagree	Slightly disagree	Slightly agree	Mostly agree	Completely agree
1. Food and meals are positive elements.						
2. I am generally pleased with my food.						
3. My life in relation to food and meals is close to ideal.						
4. With regard to food, the conditions of my life are excellent.						
5. Food and meals give me satisfaction in my daily life.						

How old are you?

What type is your employment?	
1. Dependent	2. Independent

What is your current workday?	
1. Full time job (45 h per week)	
2. Part-time job (less than 45 h per week)	

### Appendix A.3. Questionnaire for Adolescents

1. Please indicate how often you experience the following situations (mark only one response per row):

	Never	Almost never	Sometimes	Often	Always
1. How often do your parents keep track of the quantity of sweets (ice cream, cake, pastries, chocolates, etc.) that you eat?					
2. How often do your parents keep track of the quantity of snack food (potato chips, Doritos, etc.) that you eat?					
3. How often do your parents keep track of the quantity of fatty foods (hamburgers, mayonnaise, etc.) that you eat?					
4. How often do your parents keep track of the quantity of sweet drinks (soft drinks, juices) that you drink?					

2. Indicate the degree of agreement or disagreement with the following statements (mark only one answer for each row):

	Completely disagree	Mostly disagree	Slightly disagree	Slightly agree	Mostly agree	Completely agree
1. Food and meals are positive elements.						
2. I am generally pleased with my food.						
3. My life in relation to food and meals is close to ideal.						
4. With regard to food, the conditions of my life are excellent.						
5. Food and meals give me satisfaction in my daily life.						

How old are you?		
Gender	1. Male	2. Female
